# Feasibility and acceptability of novel functional electronic stimulated rehabilitation application for treatment in patients with cerebrovascular disorders: the FRAT study protocol

**DOI:** 10.1186/s40814-022-01217-7

**Published:** 2022-12-14

**Authors:** Tomoo Mano, Kiyoshi Asada, Shota Suzuki, Shu Kasama, Kaoru Kinugawa, Kazuma Sugie, Masato Kasahara, Akira Kido

**Affiliations:** 1 Department of Rehabilitation Medicine, Nara Prefecture General Medical Center, Nara, 630-8581 Japan; 2 Department of Neurology, Nara Medical University, Kashihara, 634-8521 Japan; 3 Department of Rehabilitation Medicine, Nara Medical University, Kashihara, 634-8521 Japan; 4 Institute for Clinical and Translational Science, Nara Medical University Hospital, Kashihara, 634-8522 Japan

**Keywords:** Cerebrovascular disorders, Rehabilitation, Adherence, Compression, Feasibility

## Abstract

**Background:**

The prognosis of patients with cerebrovascular disorders is poor owing to their high residual rate of hemiplegia. Delayed withdrawal from synkinesis is a major cause of prolonged hemiplegia; however, effective rehabilitation has not been established. This single-arm, open-label study aims to evaluate the influence of a low-frequency treatment device on canceling synkinesis in patients with incomplete paralysis and cerebrovascular disorders.

**Methods:**

Eligible participants will include patients aged 20 years or older with incomplete paralysis, defined as upper limb Brunnstrom stage (BRS) of 2–4, who are within 1 month of onset of a cerebrovascular disorder. Qualified patients will be assigned to the novel rehabilitation treatment with IVES+ for 4 weeks. The primary endpoint of the study is the change from baseline in the upper-limb Fugl-Meyer Assessment (FMA) 2 weeks after the start of treatment. The secondary endpoints are changes in the amount of Functional Independence Measure, changes in the amount of upper-limb BRS, and changes in the amount of Barthel Index (BI) compared to the pre-intervention value at weeks 2 and 4; changes in the upper-limb FMA scores at 1, 3, and 4 weeks; changes in grip strength compared to the pre-intervention values at 1, 2, 3, and 4 weeks; and changes in upper-limb strength (manual muscle test) compared to the pre-intervention values at 1, 2, 3, and 4 weeks.

**Discussion:**

This study will explore the usefulness of IVES+ for recovery from motor paralysis in patients with cerebrovascular disorders.

**Trial registration:**

Japanese Clinical Registry, jRCTs052180226. Date of registration: February 1, 2022

## Key messages regarding feasibility

The protocol rehabilitation therapy in this study is a new method of handy portable low-frequency treatment device, integrated volitional control electrical stimulation (IVES+), that placed the stimulating electrodes in antagonistic muscles.

- The enrolment for the patients defined as upper limb Brunnstrom stage (BRS) of 2–4 is critical to evaluating the efficacy of this study.
- This study will explore the usefulness of IVES+ in recovery from motor paralysis to cancel synkinesis in patients with cerebrovascular disorders.
- The duration of hospitalization for rehabilitation is limited, and long-term efficacy is difficult to evaluate.

## Background

In cerebrovascular disease, which encompasses various clinical conditions with very different functional outcomes, one of the sequelae of motor impairment is residual hemiplegia [1]. Not all patients reach the end of the recovery process of cerebrovascular disorders from flaccidity to associative response to synergistic and then dissociative movements; recovery often ends in the middle, and functional impairment remains [2]. Clinical experience suggests that one of the major reasons for the high residual rate of hemiplegia and decreased activities of daily living (ADL) is the delayed withdrawal of synkinesis during the recovery from cerebrovascular accidents.

One of the characteristic outcomes of cerebrovascular disorders is the unintended activation of one limb when the homologous part of the opposite limb is active. Global synkinesis, also known as mirror movement, motor overflow, and contralateral irradiation, has long been documented [3]. Global synkinesis appears in multiple joints after the acquisition of muscle contractions when routine rehabilitation treatment is performed in patients with moderate hemiplegia caused by cerebrovascular accidents. After withdrawal from synkinesis, single-joint and isolated movements become possible, and recovery from motor paralysis progresses. In general, the potential improvement period of motor paralysis after a cerebrovascular accident is within 3 months [4], and early withdrawal from synkinesis in the acute phase is important for the recovery of motor paralysis. Brain images reveal that the presence of global synkinesis involves bilateral excitation of the motor cortex, such that one hemisphere reduces its inhibitory influence on the opposite hemisphere via transcallosal fibers [5, 6]. The elbow hypertonic position associated with post-stroke is typically present during elbow flexion, although forearm pronation appears to be more common [7]. Forearm pronation is performed by the biceps brachioradialis, brachialis muscles, and pronator teres.

The rehabilitation approach encourages practitioners to “make use of the remaining function,” that is, to “acquire activities of daily living by actively enhancing the function of the non-paralyzed side and compensating for the paralyzed limb [8].” However, with the development of neuroimaging of the central nervous system, the concept of neurorehabilitation has been proposed to promote the recovery of neural function after injury. Moving paralyzed muscles through the motor learning mechanism of the brain promotes cranial nerve reconstruction. Functional electrical stimulation (FES) is one approach to this neurorehabilitation [9]. The low-frequency treatment device, integrated volitional control electrical stimulation (IVES+) (OG Giken, Tokyo, Japan), is widely used to induce and enhance muscle contraction [10, 11]. IVES+ can be applied to small muscles, for which it is difficult to locate both the stimulation and recording electrodes [12]. However, the conventional application of this device for motor paralysis, which involves only direct stimulation, is not used for the purpose of canceling global synkinesis. We believe that a new method of FES in which stimulating electrodes are placed in antagonistic muscles can release global synkinesis. The protocol of this study was designed to assess global synkinesis inhibition.

## Methods

### Design

**Fig. 1:**
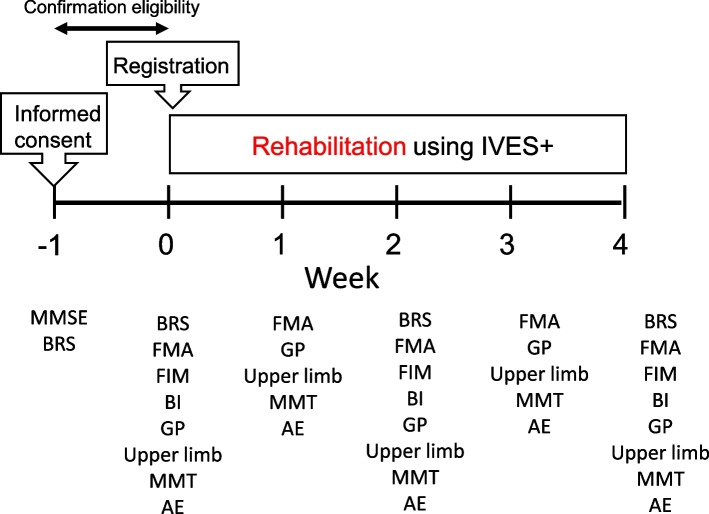
Study design. Twenty patients with incomplete paralysis and cerebrovascular disorders participated in this study. After confirmation of eligibility, the enrolled patients will be assigned to the study rehabilitation using IVES+. MMSE, Mini-Mental State Examination; BRS, Brunnstrom stage; FMA, Fugl-Meyer Assessment; FIM, Functional Independence Measure; BI, Barthel Index; MMT, manual muscle test, Adverse event; AE

Reporting was in accordance with the checklist for pilot and feasibility trials [13]. This single-arm study was designed to explore the feasibility and clinical usefulness of a novel low-frequency device for canceling global synkinesis in patients with incomplete paralysis and cerebrovascular disorders (Fig. 1). In this study, Fugl-Meyer Assessment (FMA) will be used to evaluate the degree of synkinesis cancelation. FES has been used in conventional treatments to induce and enhance muscle contraction; however, in this study, FES will be used for patients with incomplete paralysis, defined as upper-limb Brunnstrom stages (BRS) 2–4, caused by a cerebrovascular accident. Eligible participants are patients aged ≥ 20 years with moderate hemiplegia caused by a cerebrovascular accident. The inclusion and exclusion criteria are listed in Table 1. Qualified patients will undergo a novel rehabilitation program using a low-frequency treatment device and regular rehabilitation for 4 weeks (Table 2).

**Table 1:** Inclusion, exclusion, and discontinuation criteria

Inclusion criteria	
1. Within 1 month of a cerebrovascular event (cerebral infarction, cerebral hemorrhage, subarachnoid hemorrhage).	
2. With upper limb Brunnstrom stage (BRS) 2–4.	
3. Aged 20 years or older at the time of obtaining informed consent.	
4. Provides written informed consent to participate in this clinical study.	
Exclusion criteria	
1. Received treatments, such as botulinum therapy, repeated transcranial magnetic stimulation, and transcranial direct current stimulation, within 90 days of obtaining consent.	
2. With a history of surgery (including device therapy) or intravenous t-PA for a cerebrovascular event (cerebral infarction, cerebral hemorrhage, subarachnoid hemorrhage).	
3. Cognitive decline (Mini-Mental State Examination (MMSE) is 21 or less).	
4. Severe skin symptoms on the affected upper limb.	
5. Have a history of epileptic seizures.	
6. With a history of substance abuse or addiction (including alcoholism) at the time of enrolment and within the past year, or patients with complications.	
7. Using an implantable cardiac stimulator, such as a cardiac pacemaker or an implantable assisted heart.	
8. Using deep brain stimulation.	
9. With metal (excluding titanium products) implanted in the affected upper limb.	
10. Who are pregnant or may become pregnant. 11. Who are judged by doctors to be inappropriate as research subjects.	
Discontinuation criteria	
1. There was an offer to decline study participation or withdraw consent.	
2. Who were found not to meet eligibility after enrolment.	
3. The primary disease was completely cured and continued use was no longer necessary.	
4. The device therapy used in this study was judged unfavorable due to exacerbation of the primary disease.	
5. It is difficult to continue the study due to exacerbation of complications.	
6. It is difficult to continue the study due to illness.	
7. Although the frequency of use has been reduced according to the protocol, it is difficult to use even if the lower limit (less than 70% of the total planned number of uses) is reached.	
8. Who became pregnant.	
9. Poor compliance (if it is judged that less than 70% of the total planned number of uses will be used).	
10. Who cannot receive intervention due to hospital transfer.	
11. The entire study was discontinued	
12. For other reasons, the doctor judged it appropriate to discontinue the study.

**Table 2:**
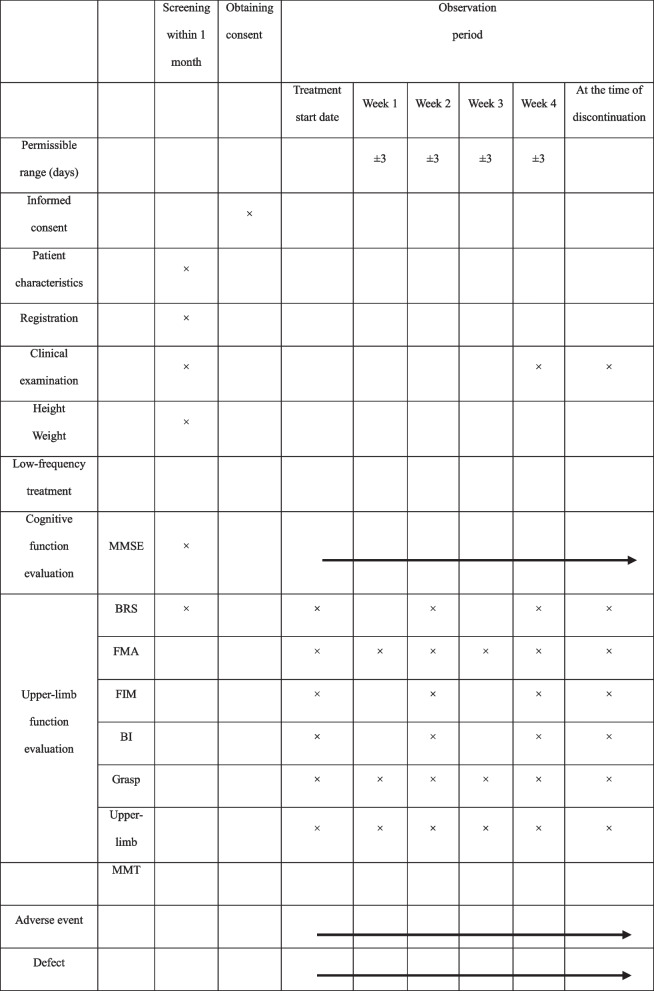
Overview of observation, clinical examination, and evaluation

*MMSE* Mini-Mental State Examination, *BRS* Brunnstrom stage, *FMA* Fugl-Meyer Assessment, *FIM* Functional Independence Measure, *BI* Barthel Index, *MMT* manual muscle test

This study will be carried out in compliance with the Declaration of Helsinki (revised in October 2013) and the Clinical Trial Act by the Ministry of Health, Labor, and Welfare in Japan. The investigator will provide sufficient explanation of the study to each patient and obtain written informed consent. The study protocol was approved by the Certified Review Board (Nara Medical University Certified Review Board: CRB5200002), and the research period is planned for February 1, 2022 to March 31, 2023. The organization of the study is shown in jRCTs052210163.

## Intervention

**Fig. 2:**
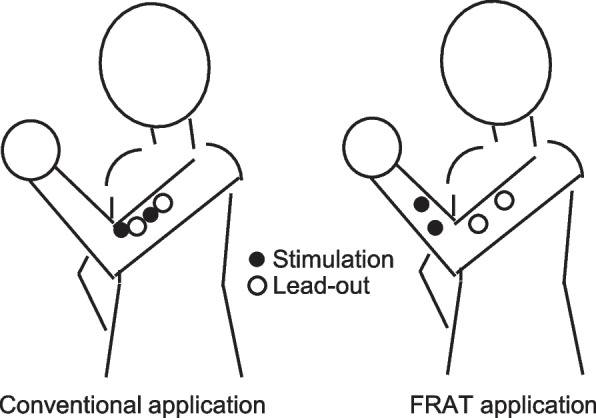
Difference between conventional application and FRAT application of IVES+. The conventional application of this device for motor paralysis is only direct stimulation, in which the lead-out and stimulation electrodes are placed on the same muscle. In this FRAT study, a lead-out electrode was placed on a muscle capable of voluntary contraction, and its contraction was monitored. Subsequently, a stimulation electrode was placed on an antagonistic muscle against a muscle that causes synkinesis

The major conventional method of using low-frequency treatment device for motor paralysis, which aims to induce and enhance the effect of muscle contraction, is the direct stimulation of the derived electrode and the stimulation electrode to the same muscle. In this study, we used IVES+, which can automatically change its stimulation intensity in direct proportion to changes in voluntarily generated electromyography (EMG) amplitude recorded with surface electrodes placed on the target muscle. In the novel FES, the derived electrode is placed on a muscle capable of voluntary contraction, and muscle activity is monitored. Next, a stimulation electrode is placed on the antagonistic muscle, which causes global synkinesis, and forced muscle contraction is promoted to perform motor control that decomposes global synkinesis (Fig. 2). FRAT application might cancel synkinesis and may help patients with cerebrovascular disorders withdraw from synkinesis at an earlier stage.

The distinctiveness of this intervention is that it is not primarily intended for strengthening muscles. Instead, it is used to retrain the neuromuscular system to execute tasks that cannot be performed voluntarily. The patients will undergo the novel FES training 20 min a day for 5 days, for 4 weeks, for a total of approximately 20 sessions, and the number of repetitions will be determined based on individual participants’ strength and endurance. The number of standard rehabilitation sessions is, in principle, fixed for all subjects but varies depending on the condition.

### Endpoints

#### Primary endpoint

The primary endpoint is the mean and standard deviation (SD) for changes from baseline in the upper limb FMA at week 2. In addition, the effectiveness of this treatment will be examined by comparing the calculated mean value with 3.0, which is estimated as the amount of change in the case of conventional treatment. The FMA scale was developed as the first quantitative evaluative instrument for measuring sensorimotor stroke recovery in patients with hemiplegic stroke. The FMA is an efficient clinical examination method that has been widely tested in stroke populations. Its primary value is the 66-point motor domain, which has received the most extensive evaluation of upper-limb function [14]. The change in the FMA score can be used to evaluate the degree of cancelation of synkinesis. FMA has the advantage of being able to capture the patient’s overall picture and making it easier to predict the effects of rehabilitation [15, 16]. FMA has been reported to correlate with the degree of independence in activities of daily living [17]. In Japanese hospitals, the general length of stay in an acute care hospital is around 2 weeks; therefore, the timing of the endpoint was planned. We planned to conduct a subgroup analysis to adjust for the number of trials after this study.

#### Secondary endpoint

For each endpoint (points 1-6 provided below), we will perform the same analysis as the primary endpoint at each evaluation time point after starting treatment from baseline.

1. Change in upper limb BRS at weeks 2 and 4,
2. Amount of change in Functional Independence Measure (FIM) at weeks 2 and 4,
3. Change in upper limb FMA at weeks 1, 3, and 4,
4. Amount of change in BI at weeks 2 and 4,
5. Amount of change in grip strength at 1, 2, 3, and 4 weeks
6. Upper limb manual muscle strength test: the amount of change in manual muscle test (MMT) at weeks 1, 2, 3, and 4.

For implementation compliance, the treatment implementation status for each day will be aggregated.

The BRS classifies the recovery process into six stages. This classification, established from clinical observations of many hemiplegic patients, is based on the degree of spasticity, synergy, and voluntary movement. A previous study reported that this classification of BRS has face validity, but it has never been assessed for reliability and validity [18]. In clinical practice, BRS is used for simple evaluation of motor paralysis, but FMA is used for more detailed evaluations. FMA is rated higher than BRS for its validity and reliability. Therefore, for the purpose of this study, we will be using both outcome measures.

The BI, which is used as a simple and useful evaluation of a patient’s independence, can be accurately and quickly scored by adhering to the definition of 10 items concerning ADL, with a score range of 0–100. The FIM is considered more valid than BI and equally reliable in the assessment of disability [19]. The FIM was developed for precise evaluation of ADL [20] and is divided into two sections: a motor section, which includes 13 items, and a cognitive section, which includes 5 items. Each item is graded from 1 (totally dependent) to 7 (completely independent), and the total FIM score range is 18–126. BI scores have been closely correlated with FIM [21]. Both will be used in this study because of the limitations of using only one assessment of ADL.

### Sample size

Since this is an exploratory study, the target number of cases is set at 20 from the feasibility viewpoint. At this time, if the average ± SD of the change amount of the main endpoint upper-limb FMA is estimated to be 6.0 ± 2.0, with reference to a previous report [22], the width of the 95% confidence interval of the change amount is approximately 1.9. As the amount of change in the case of normal treatment is expected to be 3.0 ± 1.0, it is considered that the effect of this treatment can be sufficiently confirmed.

### Statistical analysis

#### Characteristics of background and baseline values

Summary statistics (number of cases, mean, standard deviation, minimum, median, and maximum) will be reported for background and baseline distributions and continuous values in FAS, and frequency and percentage will be recorded for discrete values.

#### Statistical analysis plane

The primary endpoint is the change in the amount of upper-limb FMA from baseline to week 2. For efficacy evaluation, the full analysis set (FAS) and per-protocol set (PPS) will be used. The FAS is defined as the patient population excluded from the SS for any of the following reasons: ineligible cases after enrolment and cases with no data related to efficacy at any time after the start of study treatment. The PPS is defined as the patient population excluded from the FAS for any of the following reasons: violation of safety-related exclusion criteria, met criteria for study discontinuation, violation of effectiveness-related inclusion criteria, use of prohibited treatment, study rehabilitation compliance rate < 70%, or treatment period < 1 week.

Concerning the primary and secondary endpoints, the degree of improvement in parameters before and after intervention will be compared using the paired *t* test with a significance level of 0.05 and a tendency level of 0.10, and the improvement will be estimated using 95% CI. If the improvement in FMA between before and after the intervention is ≤ 0.10, we will proceed to the next pivotal clinical trial. Similarly, we will consider progression to the next trial if the BRS, FIM, grip strength, and BI values are ≤ 0.10. Missing values in the efficacy analysis will be supplemented with the immediately preceding value (last observation carried forward). We will analyze covariance with the intervention value as a covariate, and calculations will be performed using Statistical Package for the Social Sciences 22.0J (SPSS Japan, Tokyo, Japan).

#### Safety assessment

We will create a list of adverse events (AE) and calculate the frequency and rate of occurrence. AE will be collected for safety analysis. AEs are defined as any unfavorable or unintended sign, symptom, or disease, including abnormal laboratory values. Serious AEs include death, adverse events requiring hospitalization or prolonged hospitalization, life-threatening adverse events, and adverse events resulting in permanent or significant disability or defects. Other serious medical events based on good medical judgment may also be considered serious AEs when the patient's health is at risk and intervention is required to prevent the mentioned consequences. The safety analysis set (SS) will include patients with safety evaluation data after starting study treatment.

#### Data monitoring

All data will be stored in a database in a locked cabinet at the laboratory of the Department of Rehabilitation Medicine at Nara Medical University. The assessors will enter the data into the database system. Trial enrolment and duration will be regulated to ensure the robustness of the data.

#### The quality and safety of monitoring

Safety monitoring of this trial will be conducted by an independent physician with relevant expertise once a month. All participants and visits will be monitored by a third party.

On the other hand, the auditors will inspect several times during the period.

To increase the reliability of the evaluation, one neurological professional will perform all assessments in this study.

#### Confidentiality

All personal information about potential and enrolled participants and proxies will be stored in a manner the third party cannot access.

## Discussion

We planned this study to evaluate the effect of FES, a low-frequency treatment device, on the cancellation of synkinesis in patients with incomplete paralysis due to cerebrovascular accidents. Withdrawal from synkinesis is important for the recovery of motor paralysis, and in addition to conventional rehabilitation, canceling synkinesis using FES will likely facilitate recovery of motor paralysis in patients with moderate hemiplegia due to cerebrovascular accidents. The mechanism of FES is based on the principles of neuroplasticity [22, 23]. The recovery of motor function depends on the integrity of the neural network in the cortex of the damaged hemisphere. This is probably attributed, in part, to the temporal dynamics of corticomuscular interactions in post-stroke recovery. Especially, the changes in corticomuscular interaction are more obvious in the acute stage of stroke than in the chronic stage [24].

Since synkinesis does not appear in patients with complete paralysis with a BRS of 1 but does appear in patients with incomplete paralysis with an upper limb BRS of 2–4, we planned this cohort for enrolment. We will evaluate the usefulness of the IVES+ medical device in patients with a BRS of 2–4 using a novel method to cancel synkinesis in the early phase of cerebrovascular disorders. The level of global synkinesis intensity in the paretic arm is related to the functional outcome of patients with post-stroke hemiparesis and is particularly dependent on elbow flexor muscle contractions [25, 26]. Therefore, we will use the FES to perform dorsiflexion of the wrist when the elbow is flexed.

In this exploratory study, we will evaluate the usefulness of the medical device IVES+ for canceling global synkinesis and recovery from motor paralysis in patients with moderate hemiplegia caused by cerebrovascular disorders. This study also explores whether improved global synergy is associated with improved hand impairment. This study has the following limitations: it is a single-arm study with a short observation period and a small number of planned patients, but the result from this study will be useful for the next validation research. The main objectives of this exploratory clinical trial are feasibility and safety. Efficacy is not based on inter-group comparisons but on evaluating response or maintenance in individual cases. The selected population can be very large in studies like this, and the sample may vary. Therefore, it is necessary to perform a subgroup analysis, such as disease type, severity, or age of onset. However, due to our small sample size, our study may not have sufficient power for this analysis. As this analysis may not have sufficient power, the results should be interpreted cautiously. Contrarily, it may be possible to infer efficacy by performing a compliance analysis based on the number of treatments.

## Abbreviations

ADL: Activities of daily living
FES: Functional electrical stimulation
IVES +: Integrated Volitional Control Electrical Stimulation
BRS: Brunnstrom stages
EMG: Electromyography
FMA: Fugl-Meyer Assessment
FIM: Functional Independence Measure
BI: Barthel Index
MMT: Manual muscle test
AE: Adverse events
SS: Safety analysis set
FAS: Full analysis set
PPS: Per-protocol set
LOCF: Last observation carried forward
